# Future directions in the treatment of pelvic fractures with abdominal organ injury: the potential of combined endovascular embolization and external fixation techniques

**DOI:** 10.3389/fmed.2025.1565758

**Published:** 2025-03-28

**Authors:** Ge Ma, Zhenpeng Di, Yonglin Wen, Chao Zhang, Huaxin Hao, Yukan Li, Yinjun Zhang

**Affiliations:** ^1^Department of Critical Care Medicine, The Third Affiliated Hospital of Gansu University of Chinese Medicine, Baiyin, China; ^2^Department of Vascular Intervention, The Third Affiliated Hospital of Gansu University of Chinese Medicine, Baiyin, China

**Keywords:** pelvic fractures, abdominal organ injuries, endovascular embolization, external fixation, combined treatment, trauma care, hemorrhage control, patient outcomes

## Abstract

Pelvic fractures with abdominal organ injuries are complex and life-threatening conditions that pose significant challenges in trauma care. Current management strategies, including external fixation and interventional radiology techniques such as embolization, have shown promise in stabilizing the pelvis and controlling hemorrhage. However, these approaches face challenges such as the lack of standardized protocols, variability in patient selection, and the need for robust multidisciplinary collaboration. Additionally, the combined use of these modalities may lead to improved outcomes, including reduced mortality and shorter hospital stays, but further research is needed to optimize their application. This review aims to comprehensively explore the potential synergies between endovascular embolization and external fixation in managing these complex injuries. It critically assesses the latest clinical evidence, identifies gaps in current practices, and proposes future directions to enhance treatment effectiveness and patient outcomes.

## 1 Introduction

Pelvic fractures are a significant subset of injuries that frequently occur during high-energy trauma, representing approximately 1.5–3% of all skeletal injuries (1). They have an alarming potential for morbidity and mortality, often compounded by associated abdominal organ injuries (2). The incidence of pelvic fractures varies widely depending on the mechanism of injury, age, and gender, with studies indicating that among older adults, particularly those over 65, the frequency of pelvic fractures can exceed 30%, primarily due to falls (3). In younger populations, motor vehicle accidents account for a substantial proportion of cases (4).

The clinical significance of pelvic fractures lies not only in the direct injuries to the pelvic ring but also in their association with life-threatening complications, including severe hemorrhage and damage to abdominal organs, which can lead to a compromise in hemodynamic status (5). Research indicates that approximately 20–30% of patients with pelvic fractures will also sustain abdominal organ injuries, significantly impacting their prognosis (6). These associated injuries involve vital organs, including the bladder, liver, spleen, and intestines, often necessitating complex and urgent management strategies to prevent secondary complications (%^7^–^10^).

The management of pelvic fractures, particularly those associated with abdominal injuries, remains challenging (11). Traditional approaches tend to prioritize stabilization of the pelvic ring while addressing individual abdominal injuries through surgical management or conservative management (12). External fixation has been a cornerstone of treatment for pelvic stabilization, particularly in hemodynamically unstable patients (5). However, studies indicate that while external fixation can effectively stabilize the pelvis, it does not directly address complications arising from associated abdominal organ injuries, which can lead to increased morbidity and prolonged hospital stays (13). Recent advancements in interventional radiology have added a new dimension to the management of these complex injuries (14). The use of embolization for controlling hemorrhage from abdominal organ injuries has shown promising outcomes in terms of decreasing the need for invasive surgical procedures and reducing overall blood loss (15). A comparative analysis published in multiple studies demonstrates that patients who receive timely intervention through embolization experience shorter hospital stays and improved long-term outcomes, indicating a shift toward more hybrid management strategies that combine external fixation with endovascular embolization (16). The term ‘intervention’ in this context primarily refers to endovascular procedures, such as endovascular embolization, which are used to control hemorrhage. Surgical management, including external fixation, are also discussed as part of the combined treatment strategy.

Despite the potential advantages of combined endovascular embolization and external fixation strategies, the literature reflects a significant variability in clinical outcomes based on the timing and methodology of interventions (5, 17). For example, some studies advocate for immediate embolization in conjunction with external fixation within the same setting, citing enhanced recovery in hemodynamically unstable patients (5, 18). Conversely, other research highlights the need for a tailored approach depending on the individual patient’s injury pattern, suggesting that rigid adherence to combined strategies may not be appropriate for all cases (17).

The objective of this review is to explore the potential synergies between endovascular embolization and external fixation in managing pelvic fractures alongside abdominal organ injuries. We aim to critically assess and synthesize existing literature that investigates the efficacy, limitations, and clinical outcomes of various treatment modalities. By drawing comparisons between studies and analyzing the consistency of results, this review seeks to enhance the understanding of best practices for this challenging clinical scenario.

## 2 Mechanism of injury

The association between pelvic fractures and abdominal organ injuries is a prevalent concern, with studies suggesting that as many as 20–30% of patients with pelvic fractures will also have concurrent abdominal injuries (17). The mechanism of injury plays a pivotal role in determining the nature and severity of these associated injuries. For example, high-energy trauma, such as that resulting from vehicular accidents, typically involves significant lateral or anteroposterior compression forces, resulting in more complex fracture patterns and a higher likelihood of organ involvement (5).

A study conducted by Demetriades et al. analyzed 1,500 pelvic fracture cases and found that 28% of patients had associated abdominal injuries, with the most commonly affected organs being the spleen and liver due to their anatomical locations and susceptibility to blunt trauma (19). The mechanism of injury in these cases is primarily due to the transfer of kinetic energy from the impact, which can cause both direct bone injuries and secondary injuries to the abdominal organs.

In terms of understanding the mechanism of injury, distinguishing between various types of pelvic fractures (e.g., stable vs. unstable) is crucial. Unstable fractures are more likely to lead to significant hemorrhagic complications and organ injuries (5). A notable investigation by Jeroukhimov et al. categorized injuries into stable and unstable groups based on vital signs, finding that unstable fractures were associated with a higher incidence of intra-abdominal organ injuries (36% vs. 12% for stable fractures) (20). This delineation emphasizes the necessity for tailored treatment planning based on the injury type and mechanism.

**Figure 1:**
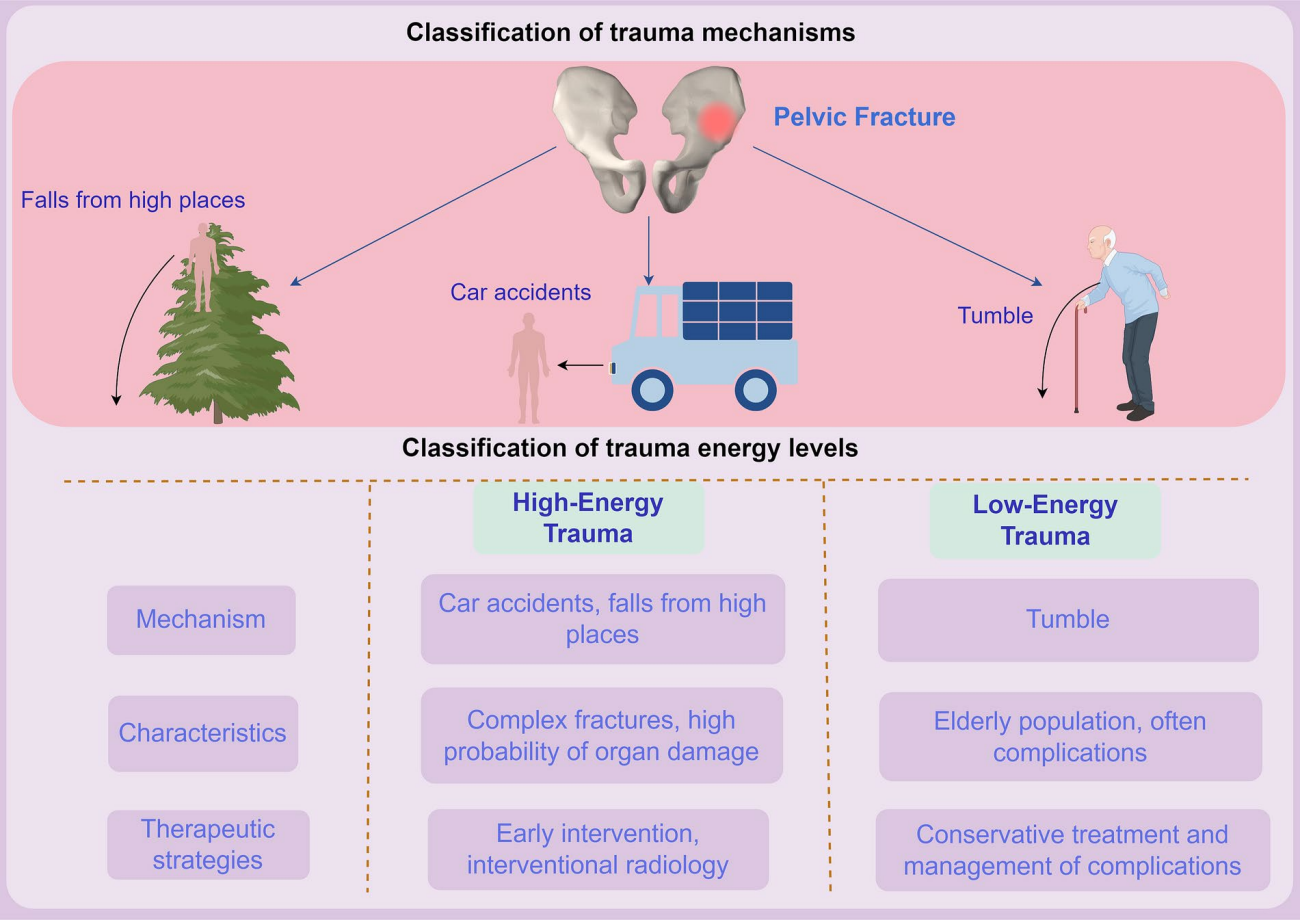
Mechanism of injury in pelvic fractures with abdominal organ injuries by Figdraw.

The relevance of understanding injury patterns extends beyond initial assessment; it significantly impacts treatment planning and outcomes (5). High-energy trauma not only results in direct bone injuries but also generates secondary injuries due to the kinetic energy transmitted through the pelvis (21) (Figure 1). For instance, when a fracture is accompanied by significant soft tissue disruption, the risk of hemorrhage and organ compromise escalates (22). Endeshaw et al. (23) highlighted that prompt recognition of associated abdominal injuries during the initial trauma assessment directly correlates with reduced morbidity and improved survival rates. Despite the established knowledge of injury patterns, substantial variability exists across studies regarding the identification and management of pelvic fractures with concurrent abdominal injuries (5). For instance, some research indicates that up to 50% of intra-abdominal injuries may go unrecognized in the initial evaluation, particularly in cases where the pelvic fracture is the primary focus (24). This underscores the importance of thorough examination protocols, including imaging modalities and interdisciplinary consultations, to ensure comprehensive evaluation and timely intervention.

In conclusion, the epidemiology of pelvic fractures and associated abdominal organ injuries reflects complex interactions between demographic factors, mechanisms of injury, and treatment implications. High-energy mechanisms, particularly those affecting younger populations, typically result in more severe injuries and higher complication rates (5). Conversely, older adults often sustain fractures from low-energy falls and frequently present with concurrent medical comorbidities, complicating their overall management (Figure 1). An acute awareness of these pattern differences is essential for developing effective treatment protocols that optimize patient outcomes (25). In light of the growing body of literature, there is a pressing need for ongoing research that not only explores the mechanisms underlying pelvic fractures but also evaluates innovative multidisciplinary approaches to enhance the care of patients facing these challenging injuries. This understanding will ultimately guide clinicians in making informed decisions that align with evidence-based practices, enhancing both immediate interventions and long-term recovery for patients suffering from these complex trauma presentations.

## 3 Current treatment paradigms for pelvic fractures with abdominal injury

The management of pelvic fractures, particularly those associated with abdominal organ injuries, represents a complex challenge in trauma care (26). Current treatment paradigms typically encompass a combination of conservative approaches and surgical management aimed at stabilizing the pelvis while addressing associated abdominal injuries (5). Understanding these management strategies is crucial for optimizing patient outcomes and minimizing complications.

Standard management approaches generally fall into two categories: conservative and surgical (27). Conservative management may include bed rest, pain control, and pelvic binding to promote stability, especially in patients with stable fractures who exhibit no clear signs of intra-abdominal injury (27). In contrast, surgical management is warranted for unstable fractures or significant associated injuries. Techniques such as external fixation, open reduction and internal fixation (ORIF), and endovascular embolization have become embedded in trauma practice (5).

Recent studies indicate that external fixation remains a primary strategy in the management of unstable pelvic fractures (28, 29). This method is particularly advantageous for patients who present with hemodynamic instability, as it provides immediate stabilization of the pelvis and can help control hemorrhaging (29). Zhao et al. highlighted the efficacy of external fixation in reducing pelvis volume, thereby decreasing the potential for further bleeding from surrounding vascular structures. Importantly, external fixation may serve as a temporizing measure while plans for definitive surgical management, such as internal fixation, are made (30).

However, despite the advantages of current management strategies, significant shortcomings persist in addressing both pelvic stability and associated abdominal injuries comprehensively (31). One major limitation of external fixation is that it does not directly address intra-abdominal injuries, which can lead to increased morbidity (28). For instance, Zhao et al. (30) found that patients receiving external fixation alone had a higher incidence of delayed abdominal complications compared to those who underwent adjunctive procedures like laparotomy or embolization. Moreover, while external fixation can stabilize the pelvic ring, it often necessitates additional surgical procedures to repair intra-abdominal injuries, prolonging hospitalization and increasing the risk of postoperative complications (30).

Another significant challenge lies in the selection of appropriate treatment based on the mechanism of injury. High-energy impacts result in complex fracture patterns that may complicate surgical planning (17). A systematic review by Sawauchi et al. revealed that patients with high-energy pelvic trauma requiring surgical management faced a 27% rate of complications, including infection, nonunion, and reoperation. Such variability highlights the necessity for an individualized approach to management and reinforces the critical need for interdisciplinary coordination between trauma surgeons, orthopedic surgeons, and interventional radiologists (5).

In light of these challenges, several case studies have recently reported on treatment outcomes and complications associated with the management of pelvic fractures and abdominal injuries. For instance, a case series by Lin et al. documented the outcomes of 15 patients with pelvic fractures complicated by splenic injuries (32). In this series, patients who underwent laparoscopic splenectomy in conjunction with external fixation exhibited reduced bleeding complications and shorter overall hospitalization compared to those managed with traditional laparotomy alone (9 days vs. 14 days, respectively) (32). These findings underscore the potential benefits of a combined approach that integrates external fixation with appropriate management of abdominal injuries. Similarly, Wendler et al. examined outcomes in a cohort of trauma patients with pelvic fractures and renal injuries (33). Patients who were treated with isolated external fixation experienced significant rates of renal complications, prompting the authors to suggest that an integrative approach involving both external fixation and endovascular embolization should be considered to mitigate the risk of renal failure (33). These results illustrate the need for additional research to optimize treatment strategies tailored to specific injury patterns.

The question of timing is also crucial in discussing current treatment paradigms. Early intervention has proven vital in addressing both pelvic stabilization and intra-abdominal organ injuries (34). Tiziani et al. (34) demonstrated that patients undergoing early intervention, which included a combination of pelvic external fixation and surgical management of intra-abdominal injuries, had a significantly lower incidence of complications (15% vs. 34%) compared to those whose treatment was delayed. Such findings highlight the pressing need for prompt recognition and intervention in trauma settings to improve patient outcomes.

The management of pelvic fractures with concomitant intestinal injuries presents a unique set of challenges. Intestinal injuries, which can range from contusions to perforations, often necessitate surgical intervention and are associated with significant morbidity and mortality. Endeshaw et al. (23) highlighted the importance of timely recognition and management of intestinal injuries in patients with pelvic fractures, as delays can lead to septic complications and increased mortality. They found that patients with delayed diagnosis of intestinal injuries had a higher incidence of intra-abdominal abscesses and longer hospital stays. In contrast, timely laparotomy and repair were associated with improved outcomes. In addition, the application of laparoscopic techniques in the setting of intestinal injuries requires careful consideration, as the presence of contamination may increase the risk of surgical site infections. Comparative studies are needed to further evaluate the optimal surgical approaches for pelvic fractures with intestinal injuries and to determine the role of minimally invasive techniques in this patient population.

In summary, while standard management approaches for pelvic fractures with abdominal injuries include a mix of conservative and surgical strategies, significant challenges remain in addressing the dual aspects of pelvic stability and organ injury comprehensively (35). Current modalities often fall short in preventing complications and ensuring positive outcomes, necessitating further research and innovation in treatment approaches. Case studies underscore the variable outcomes associated with different management strategies, reinforcing the necessity for personalized, multi-disciplinary care (27). By addressing the shortcomings of existing paradigms and advocating for combined approaches, future treatments can potentially increase stability, reduce morbidity, and enhance recovery in patients suffering from pelvic fractures complicated by abdominal organ injuries (5). Continued research and clinical trials are essential to refine these strategies, ultimately leading to improved care for this complex patient population.

## 4 Endovascular embolization in the management of pelvic fractures with abdominal organ injury

Pelvic fractures accompanied by abdominal organ injuries represent a significant challenge in trauma care, necessitating a multifaceted approach that addresses both the pelvic and abdominal components of the injury (17). Endovascular embolization has emerged as a critical component in the management of these complex injuries, particularly for controlling hemorrhage from abdominal organ injuries (36). The importance of timely intervention cannot be overstated, as several studies have demonstrated a strong correlation between reduced time to angioembolization and improved mortality outcomes in patients with severe pelvic fractures (37, 38).

### 4.1 Techniques of embolization

Endovascular embolization has gained traction as a minimally invasive method to control hemorrhage arising from pelvic fractures, particularly in patients with associated abdominal organ injuries (39). Initial studies highlighted its utility in patients with hemodynamic instability, specifically detailing the protocol for identifying the bleeding source via computed tomography (CT) before proceeding with embolization (40, 41). Recent advancements in imaging, including multi-detector CT and digital subtraction angiography, increased diagnostic accuracy and expedited the embolization process (42, 43). For instance, a randomized clinical trial compared direct retroperitoneal pelvic packing with endovascular embolization in hemodynamically unstable patients and found that embolization resulted in a significantly quicker time to intervention and lower mortality rates (44). This method is particularly favored in cases with arterial bleeding due to its specificity and minimally invasive nature (45).

### 4.2 Patient demographics and efficacy

The efficacy of endovascular embolization varies significantly across different demographic groups, particularly among the elderly, who often present with unique challenges in trauma scenarios (46). The literature suggests that elderly patients sustain pelvic fractures through lower-energy mechanisms but may still require aggressive interventions due to frailty and existing comorbidities (46, 47). For example, Morozumi et al. (48) reported a case of severe pelvic injury in an elderly patient, where both trans-arterial embolization and a comprehensive multidisciplinary approach successfully stabilized the patient. Furthermore, Yanagi et al. (49) demonstrated that patients with American Association for the Surgery of Trauma Grade 4 renal injuries experienced improved outcomes with early intervention, underscoring the need for rapid treatment protocols tailored to the patient’s age and injury pattern.

### 4.3 Comparative outcomes in trauma protocols

The evolving approach of combining interventional radiology with surgical methods is re-defining treatment paradigms. A study has explored the role of resuscitative endovascular balloon occlusion of the aorta (REBOA) in conjunction with embolization, often within a hybrid operating room environment (50). Jarvis et al. (50) emphasized that the integration of these modalities could minimize procedure time and improve logistical efficiency, particularly important for patients with concurrent trauma. While the integration of procedures like REBOA has shown promise in managing hemorrhage, Jansen et al. (51) raise concerns that REBOA may delay hemostasis and worsen survival prognosis. This viewpoint, although not universally accepted, highlights the ongoing debate in trauma care. Critics argue that REBOA’s impact on hemodynamics can be complex, potentially leading to adverse outcomes if not meticulously managed. However, proponents emphasize its utility in select cases, underscoring the need for further research to define its role clearly. As noted by Burlew et al. (52), combined strategies including REBOA can optimize care for life-threatening hemorrhage, while Metsemakers et al. (53) demonstrate the value of transcatheter embolotherapy following external fixation. These contrasting findings underscore the importance of individualized treatment protocols and the necessity for robust evidence-based guidelines.

The implementation of hybrid strategies has also shown promise in overcoming traditional treatment delays. A recent cohort study demonstrated substantial advancements in hemorrhage management and overall survival rates when employing a Trauma Hybrid Operating Room (THOR) (54). This blend of surgical exploration and interventional radiology facilitates the prompt control of hemorrhage while maximizing resource utilization across specialties (Table 1).

**Table 1:** Clinical application of interventional therapy in pelvic fracture and abdominal organ injury.

Objective	Study types	Condition	Patients (n)	Main findings	References
Evaluating the factors associated with the need for TAE in patients without CE on CT scan	Retrospective case control	Pelvic fracture	201	Relative hypotension increases the probability of the need for angioembolisation in pelvic fracture patients without contrast extravasation on computed tomography scan	Kuo et al. (37)
Effect of door-to-angioembolization time on mortality in pelvic fracture	Retrospective cohort	Pelvic fracture	181	Every hour of delay in angioembolization increases mortality in pelvic fracture patients	Matsushima et al. (38)
Prevalence of pelvic CT angiography (CTA) and angiographic embolization in geriatric patients with pelvic ring fractures presenting to two level I trauma centers	Retrospective study	Geriatric patients with pelvic ring fractures	190	Pelvic CTA and angiographic embolization are valuable tools for managing geriatric patients with pelvic ring fractures, with a significant prevalence of their use in level I trauma centers	McDonald et al. (39)
Role of multidetector-row CT in assessing the source of arterial hemorrhage in patients with pelvic vascular trauma	Comparative study	Pelvic vascular trauma	28	Multidetector-row CT is effective in assessing the source of arterial hemorrhage, with good correlation with angiography	Pinto et al. (40)
Availability of angioembolization after hours and on weekends and its impact on pelvic trauma care	Retrospective study	Pelvic trauma	191	Availability of angioembolization varies after hours and on weekends, leading to two standards of care and increased time to therapeutic intervention	Schwartz et al. (41)
The role of multidetector computed tomography versus digital subtraction angiography in triaging care and management in abdominopelvic trauma	Comparative study	Abdominopelvic trauma	51	Both CECT and DSA have roles in triaging and managing abdominopelvic trauma, with CECT providing rapid assessment and DSA offering detailed vascular imaging	Hallinan et al. (42)
Management strategy for open pelvic fractures	Retrospective observational study	Open pelvic fractures	47	A 11-year single-center study showed the management strategies and outcomes for open pelvic fractures, highlighting the importance of a multidisciplinary approach	Choi et al. (43)
Retroperitoneal packing or angioembolization for hemorrhage control of pelvic fractures	Quasi-randomized clinical trial	Hemodynamically unstable pelvic fracture patients with Injury Severity Score ≥ 33	56	Angioembolization resulted in quicker time to intervention and lower mortality rates compared to retroperitoneal packing	Li et al. (44)
Arterial embolisation for trauma patients with pelvic fractures in emergency settings	Nationwide matched cohort study	Trauma patients with pelvic fractures	17,670	The study evaluated the effectiveness of arterial embolisation in emergency settings, showing its benefits in managing hemorrhage	Furugori et al. (45)
Hemorrhage requiring embolisation after low energy pelvic fracture in an elderly patient	Case report	Low energy pelvic fracture in elderly	1	Presented a case where an elderly patient with a low energy pelvic fracture required embolisation due to hemorrhage, highlighting the unique challenges in this population	Martin and Casey (46)
Clinically relevant bleeding risk in low-energy fragility fractures of the pelvis in elderly patients	Retrospective study	Low-energy fragility fractures of the pelvis in elderly	322	Identified the clinically relevant bleeding risk in this specific patient group, emphasizing the need for tailored management strategies	de Herdt et al. (47)
Trans-arterial and trans-venous interventional radiology for an elderly patient with life-threatening pelvic injury after accidental falling due to life-threatening cardiac arrhythmia: a case report	Case report	Elderly patient with life-threatening pelvic injury	1	Successful management of life-threatening pelvic injury using trans-arterial and trans-venous interventional radiology in an	Morozumi et al. (48)
Early transcatheter arterial embolization for the American association for the surgery of trauma grade 4 blunt renal trauma in two institutions	Prospective study	Blunt renal trauma patients	190	Early transcatheter arterial embolization significantly reduces mortality and complications in patients with severe blunt renal	Yanagi et al. (49)
A descriptive survey on the use of REBOA for pelvic fractures at US level I trauma centers	Descriptive survey	Pelvic fractures	–	Provided insights into the use of REBOA in pelvic fractures across different trauma centers in the US	Jarvis et al. (50)
Emergency department resuscitative endovascular balloon occlusion of the aorta in trauma patients with exsanguinating hemorrhage: the UK-REBOA randomized clinical trial	Randomized clinical trial	Pelvic fractures with exsanguinating hemorrhage	190	REBOA may delay hemostasis and worsen survival prognosis	Jansen et al. (51)
Preperitoneal pelvic packing/external fixation with secondary angioembolization: optimal care for life-threatening hemorrhage from unstable pelvic fractures	Retrospective review	Unstable pelvic fractures	62	This approach effectively controls hemorrhage and stabilizes the pelvis, allowing for better intra-abdominal surgical access	Burlew et al. (52)
Transcatheter embolotherapy after external surgical stabilization is a valuable treatment algorithm for patients with persistent hemorrhage from unstable pelvic fractures: outcomes of a single center experience	Single center experience	Unstable pelvic fractures with persistent hemorrhage	803	Transcatheter embolotherapy after external fixation is a valuable treatment algorithm for patients with persistent hemorrhage	Metsemakers et al. (53)
THOR shortened procedure time in abdominopelvic trauma patients requiring surgery and interventional radiology procedures	Cohort study	Abdominopelvic trauma	91	Demonstrated that the use of THOR can significantly shorten procedure time for patients requiring both surgery and interventional radiology procedures	Prichayudh et al. (54)

CE, contrast extravasation; TAE, transcatheter arterial embolisation; CT, computed tomography; CECT, contrast-enhanced computed tomography; DSA, digital subtraction angiography; REBOA, resuscitative endovascular balloon occlusion of the aorta; THOR, Trauma Hybrid Operating Room.

## 5 External fixation: current role and innovations

External fixation has emerged as a pivotal treatment modality for unstable pelvic fractures, providing stabilization and facilitating early mobilization while minimizing visceral organ compromise. Recent advancements in technique and technology have further defined the role of external fixation in trauma surgery, particularly in the context of pelvic fractures with abdominal organ injuries (55). This section critically examines the current applications of external fixation while highlighting relevant innovations and study comparisons.

### 5.1 The role of external fixation in treating pelvic fractures

The stability provided by external fixators is crucial in the management of unstable pelvic fractures, especially when associated with abdominal organ injuries. Hu et al. (55) demonstrated that early application of external fixation significantly reduces morbidity associated with these injuries, providing immediate stabilization that can facilitate subsequent interventions such as embolization or surgical fixation. Furthermore, Stewart et al. (56) carried out a systematic review and meta-analysis that affirmed the effectiveness of external fixation in promoting hemodynamic stability and preventing the complications associated with internal fixation, especially in the context of hemodynamically unstable pelvic fractures.

### 5.2 Innovations and evolving techniques in external fixation

Recent innovations have sought to enhance the effectiveness and safety of external fixation techniques. The anterior pre-tensioned external fixator proposed by Queipo-de-Llano et al. marks a significant advance in providing customizability to external fixation, potentially enhancing stability while decreasing soft tissue complications (57). Additionally, a study explored supra-acetabular external fixation, providing data indicative of its effectiveness through a digital anatomical study, which elucidated optimal pin placement and its implications for pelvic stability. These innovations are aligned with the growing recognition that proper alignment and stabilization of the pelvic ring are critical in improving overall patient outcomes (58). The role of specific fixation sites in relation to pelvic anatomy should be an essential consideration in future practice, as indicated by the lateral posterior fixation techniques described by Russ et al., which propose alternative pin sites to avoid soft tissue damage (59).

### 5.3 Comparative efficacy of external fixation

Although external fixation is generally accepted as a valid treatment for unstable pelvic fractures, variability in patient outcomes necessitates a comprehensive understanding of its comparative efficacy (60). Various studies have yielded differing conclusions on the optimal fixation techniques. For example, Kim et al. (61) noted significant differences in mortality associated with different hemorrhage-control methods used in conjunction with external fixation, pointing to the critical interaction between the fixation technique and overall patient management.

Moreover, a compelling analysis by Ohmori et al. established that patients treated with external fixation exhibited improved survival rates compared to those who did not receive such intervention, emphasizing the mortality benefit afforded by effective stabilization (62). However, Schmal et al. (63) noted higher complication rates with external fixation when compared to primary stabilization methods like C-clamp application, thus calling for a careful assessment of techniques based on specific injury profiles. In examining the various approaches, Ma et al. (64) compared interval fixation with external fixation and found that while both techniques provided adequate results, external fixation generally posed fewer soft tissue complications, often a determinant of successful surgical outcomes. Such discrepancies necessitate a thorough evaluation of individual clinical scenarios to define the optimal fixation strategy tailored to patient needs.

In summary, external fixation plays an essential role in the management of unstable pelvic fractures, evidenced by its benefits in stabilization and reduction of complications (65). Innovations in external fixation techniques and the integration of concurrent intervention strategies have the potential to optimize treatment outcomes (65). Future research must continue to refine these practices, aligning with the dual goals of maximizing efficacy and minimizing complications in trauma care. As the landscape of pelvic fracture management evolves, external fixation will undoubtedly remain pivotal in addressing these complex clinical challenges while advancing patient care (Table 2).

**Table 2:** Studies on the application of external fixation to pelvic fractures.

Objective	Study types	Condition	Patients (n)	Main findings	References
The role of external fixation in treating pelvic fractures	Systematic review and meta-analysis	Unstable pelvic fractures	32	Early application of external fixation significantly reduces morbidity associated with these injuries, providing immediate stabilization that can facilitate subsequent interventions such as embolization or surgical fixation	Hu et al. (55)
The effectiveness of external fixation in promoting hemodynamic stability and preventing complications associated with internal fixation	Systematic review and meta-analysis	Hemodynamically unstable pelvic fractures	539	External fixation is effective in promoting hemodynamic stability and preventing complications associated with internal fixation	Stewart et al. (56)
The role of anterior pre-tensioned external fixator in pelvic fractures and dislocations	Initial clinical series	Pelvic fractures and dislocations	13	The anterior pre-tensioned external fixator provides customizability to external fixation, potentially enhancing stability while decreasing soft tissue complications	Queipo-de-Llano et al. (57)
Supra-acetabular external fixation for pelvic fractures: a digital anatomical study	Digital anatomical study	Pelvic fractures	120	The study elucidates optimal pin placement and its implications for pelvic stability	Wang et al. (58)
An alternative site for pin placement in external fixation of pelvic fractures	Surgical technique	Pelvic fractures	27	Proposes alternative pin sites to avoid soft tissue damage	Russ et al. (59)
Variability in pelvic packing practices for hemodynamically unstable pelvic fractures at US level I trauma centers	Retrospective study	Hemodynamically unstable pelvic fractures	190	External fixation is associated with lower complication rates compared to primary stabilization methods like C-clamp application	Blondeau et al. (60)
Comparison of mortality among hemorrhage-control methods performed for hemodynamically unstable patients with traumatic pelvic fractures	Multi-center study	Hemodynamically unstable patients with traumatic pelvic fractures	97	Significant differences in mortality associated with different hemorrhage-control methods used in conjunction with external fixation	Kim et al. (61)
The impact of external fixation on mortality in patients with an unstable pelvic ring fracture	Propensity-matched cohort study	Unstable pelvic ring fractures	1,163	Patients treated with external fixation exhibited improved survival rates	Ohmori et al. (62)
Effectiveness and complications of primary C-clamp stabilization or external fixation for unstable pelvic fractures	Comparative study	Unstable pelvic fractures	5,499	Higher complication rates with external fixation when compared to primary stabilization methods like C-clamp application	Schmal et al. (63)
Interval versus external fixation for the treatment of pelvic fractures	Comparative study	Pelvic fractures	263	External fixation generally poses fewer soft tissue complications	Ma et al. (64)

## 6 Combined approach: the synergy of endovascular embolization and external fixation

**Figure 2:**
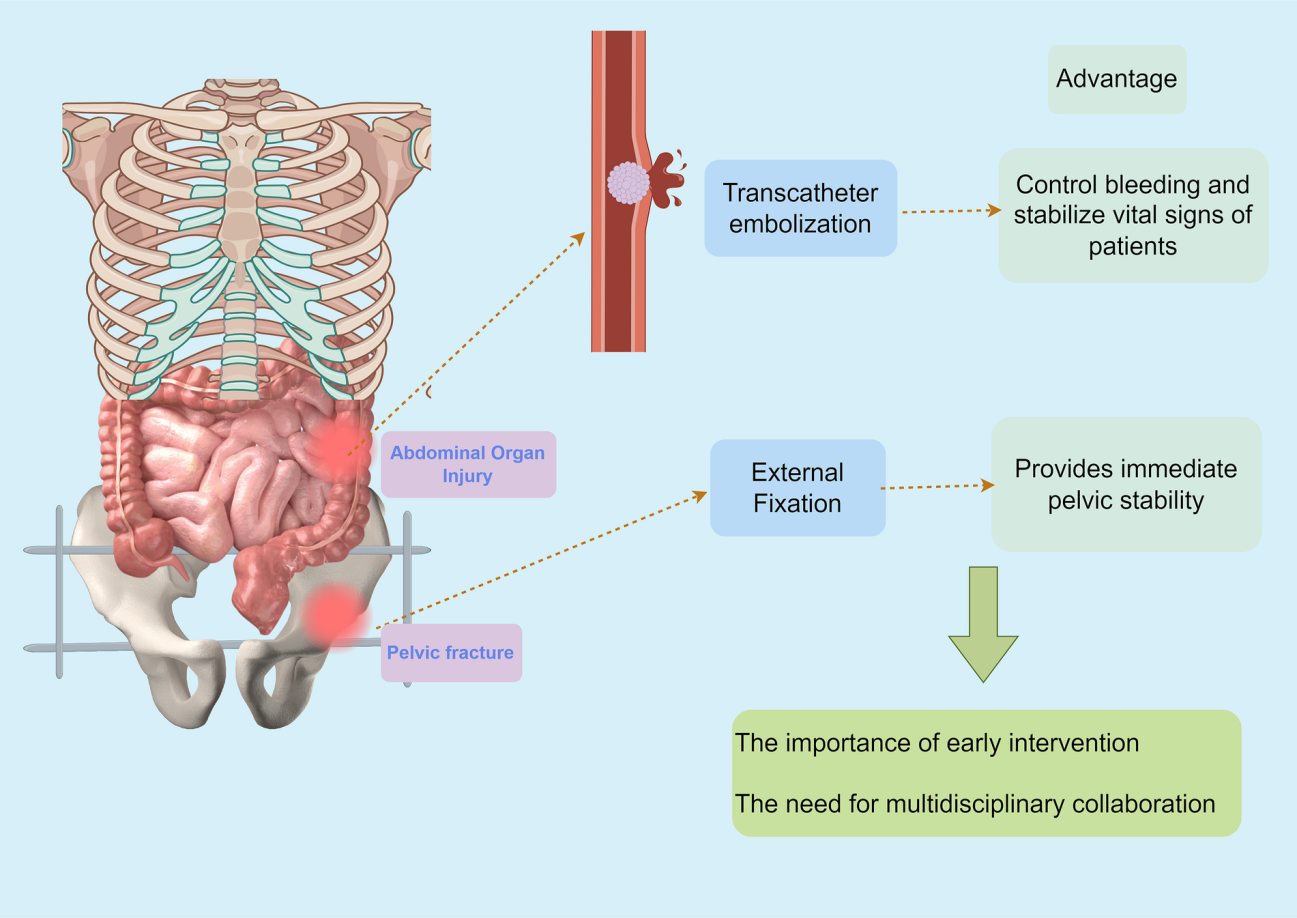
Combined approach: the synergy of interventional and external fixation by Figdraw.

The management of pelvic fractures accompanied by abdominal organ injury presents a unique challenge in trauma surgery (19). The synergy of endovascular embolization and external fixation has shown great promise in optimizing patient outcomes, particularly in hemodynamically unstable cases (66) (Figure 2). The availability of advanced techniques allows for a tailored approach, enhancing hemorrhagic control while stabilizing the pelvis.

### 6.1 The rationale for a combined approach

This integrated approach leverages the strengths of both modalities to enhance hemorrhage control and pelvic stability. The use of external fixation provides immediate mechanical support to the fractured pelvis, reducing further injury and facilitating early mobilization (53). Meanwhile, endovascular embolization offers a minimally invasive method to control hemorrhage, particularly from arterial sources, which is often difficult to achieve with surgical methods alone. This combination not only addresses the immediate life-threatening hemorrhage but also stabilizes the pelvis, allowing for better intra-abdominal surgical access and reducing the risk of secondary complications (62).

The combined approach is further justified by the high incidence of intra-abdominal organ injuries associated with pelvic fractures. Studies have shown that up to 30% of patients with pelvic fractures sustain concomitant abdominal injuries, often involving critical organs such as the bladder, liver, spleen, and intestines (19, 20, 53). These injuries require urgent and often complex management strategies to prevent secondary complications and improve patient outcomes. The integrated use of external fixation and endovascular embolization allows for a more comprehensive treatment plan that addresses both the pelvic and abdominal components of the injury simultaneously. This approach has been shown to reduce mortality rates and improve overall patient outcomes, particularly in patients with severe hemodynamic instability (53). Several studies have highlighted the critical importance of timely intervention in cases of unstable pelvic fractures with associated hemorrhage (5). Burlew et al. (52) recommend a combined strategy of preperitoneal pelvic packing followed by external fixation and secondary angioembolization when managing life-threatening hemorrhage from unstable pelvic fractures. This approach not only controls hemorrhage effectively but also stabilizes the pelvic anatomy, allowing for better intra-abdominal surgical access (52) (Figure 2). Marzi and Lustenberger (67) emphasized the management of bleeding pelvic fractures, noting that an integrated approach improves control over hemorrhage and ultimately contributes to better survival rates.

### 6.2 Efficacy of external fixation and angioembolization

External fixation serves as a crucial component of the combined approach, offering immediate mechanical stability to the pelvic fracture while allowing for ongoing assessment and management of hemorrhage (30) (Figure 2). Ohmori et al. (62) conducted a propensity-matched cohort study that indicated significant reductions in mortality for patients who received external fixation in conjunction with other treatment modalities. Similarly, Metsemakers et al. (53) found that transcatheter embolotherapy following external fixation plays a valuable role in managing persistent hemorrhage, thereby enhancing patient outcomes.

While these studies support the effectiveness of combined modalities, it is important to discuss the variability in outcomes based on different surgical experiences and institutional protocols. For instance, the effectiveness can be influenced by the timing of interventions. Early intervention, as observed in studies such as that of Kim et al., shows a correlation with improved mortality outcomes in patients undergoing hemostatic control techniques, including external fixation (61). In contrast, Tanizaki et al. (68) demonstrates that early embolization without external fixation can still be effective, but may not achieve the same level of stabilization.

### 6.3 Complications and considerations

A crucial aspect to consider in implementing a combined approach is the potential for complications it may introduce. Baker et al. (69) identified risk factors associated with pelvic infections following pre-peritoneal packing, stressing the need for optimal surgical technique and patient selection to mitigate such risks. Additionally, excessive or inappropriate fixation may lead to restenosis or complications related to the fixation device itself, underscoring the need for careful planning and execution during surgery.

Despite these concerns, the consensus remains that a combined strategy offers the best outcomes for managing hemodynamically unstable pelvic fractures. Perumal et al. (70) suggested further research into risk stratification and protocol standardization could aid in optimizing patient selection for this approach. Furthermore, the role of multidisciplinary collaboration in managing complex trauma cases is emphasized, as various specialties can provide valuable insights into patient care (71).

### 6.4 Future directions in combined interventional strategies

Looking forward, further studies are needed to refine and standardize intervention protocols that utilize both external fixation and interventional radiology techniques. Insights from the most recent systematic reviews, such as those by Zheng et al. exploring hemostatic interventions, suggest that an integrative approach involving advanced imaging and timely intervention could greatly improve outcomes in this patient population (72). Incorporating new technologies like point-of-care ultrasound could assist in the assessment of pelvic stability and bleeding sources, paving the way for a more dynamic response strategy.

In summary, the combined approach of endovascular embolization and external fixation represents a promising evolution in the management of pelvic fractures with abdominal organ injury (73). The synergy of these modalities enhances hemorrhage control, provides mechanical stability, and improves overall survival probabilities, albeit with considerations toward possible complications and the necessity for further research (74). The integration of multidisciplinary teams and refined protocols will be essential in ensuring optimal outcomes as we advance in trauma care technologies and methodologies.

## 7 Challenges and future directions

While the combined approach to treating pelvic fractures with abdominal organ injury shows promise, it is encumbered by various challenges that necessitate further exploration and standardization in clinical practice. Collaborative efforts in research and clinical applications will be vital in realizing the full potential of such integrative strategies (75).

The implementation of combined interventional and external fixation strategies for pelvic fractures complicated by abdominal organ injury necessitates a coordinated effort among different specialties, including trauma surgery, interventional radiology, orthopedic surgery, and emergency medicine (76, 77). Fragmentation of care can hinder timely interventions, adversely affecting patient outcomes. Schwartz et al. (41) highlighted that the availability of angioembolization services outside of regular hours can significantly delay treatment, thereby increasing mortality rates in critically injured patients. This delay is particularly detrimental in cases of concurrent abdominal organ injuries, where hemorrhage from organs like the liver or spleen demands immediate intervention alongside pelvic stabilization. A recent study by Li et al. (24) emphasized that delays in addressing intra-abdominal hemorrhage in pelvic fracture patients correlate with a 2.5-fold increase in mortality, underscoring the need for synchronized protocols.

The technical complexity involved in performing both interventional procedures and external fixation poses additional challenges (37). For abdominal organ injuries, embolization efficacy varies depending on the organ involved. For instance, splenic injuries often require precise embolization to preserve parenchymal function, whereas hepatic injuries may necessitate more extensive embolization, increasing the risk of ischemic complications (9, 36). Kuo et al. (37) demonstrated that relative hypotension in pelvic fracture patients may influence the need for angioembolization, impacting the technical execution of interventions. While several protocols exist, there remains inconsistency in practice, especially regarding the criteria for selecting patients for combined interventions. For instance, differences in thresholds for patient stability prior to the adoption of embolization techniques have generated disparate outcomes in similar cases (44). A comparative analysis by Tan et al. (16) revealed that combined embolization and fixation reduced mortality in liver injury-associated pelvic fractures (15% vs. 28% with fixation alone) but showed no significant benefit in isolated splenic injuries, highlighting the need for injury-specific protocols.

The efficacy of combined treatment strategies may also be influenced by patient-specific factors, such as age, comorbidities, and the mechanism of injury (78). Abdominal organ injuries in elderly patients often involve friable vasculature and pre-existing conditions (e.g., cirrhosis or anticoagulant use), complicating embolization outcomes. Delamare et al. (79) discussed how REBOA might not yield equivalent benefits in older cohorts when compared to younger patients, owing to different hemodynamic responses and additional cardiac considerations. Furthermore, the risk of intra-abdominal hemorrhagic shock in elderly patients with low-energy pelvic fractures has been notably highlighted, calling for modified approaches that acknowledge this demographic’s unique requirements (47).

Access to appropriate imaging and interventional resources is a significant limitation, particularly in trauma centers operating within the constraints of limited resources. Rapid identification of abdominal organ injuries via contrast-enhanced CT (CECT) is critical, yet disparities in imaging availability persist. Research by Jarvis et al. underscores variability in interventional radiology availability across trauma centers, attributing delays to unavailability of specialized personnel (50). Such discrepancies can lead to adverse outcomes, particularly for patients requiring immediate embolization in urgent scenarios (80). Additionally, disparities in healthcare resource distribution challenge timely access to combined treatment modalities and can create inequality in the treatment of traumatic pelvic fractures across different regions.

The potential for adverse outcomes remains a concern with the combined treatment approach (39). For abdominal injuries, post-embolization complications such as hepatic necrosis or splenic abscess formation are underreported in pelvic fracture studies. A systematic review by Wallis et al. (15) found that 12% of patients undergoing hepatic embolization developed ischemic complications, necessitating secondary surgeries. Similarly, Martin and Casey (46) documented that patients with low-energy pelvic fractures may still require embolization despite being perceived as stable previously, suggesting that the risk of overlooked vascular injuries remains significant. Moreover, the association between angioembolization and potential complications, such as ischemic damage to surrounding tissues, must be constantly evaluated. Recently, Furugori et al. (45) highlighted that while the outcomes have improved through combined modalities, the risk–benefit ratio for each patient remains a cardinal concern. Thus, future studies must address the delineation of complications specifically attributable to combined interventions.

Furthermore, the application of combined endovascular embolization and external fixation techniques in pelvic fractures with intestinal injury deserves special attention. Intestinal injury, as one of the severe abdominal organ injuries associated with pelvic fractures, poses significant challenges in clinical management. Tanizaki et al. (68) demonstrated that early embolization without external fixation could still effectively control hemorrhage in pelvic trauma patients, but the stability provided by external fixation is crucial for preventing secondary intestinal injury and facilitating subsequent surgical interventions for intestinal repair. In contrast, some researchers argue that the combined approach may increase the risk of intra-abdominal complications, such as intestinal ischemia or infection, especially in patients with pre-existing comorbidities like cardiovascular diseases or diabetes. For instance, Li et al. (24) indicated that patients with pelvic fractures and intestinal injury who underwent combined treatment had a higher incidence of postoperative complications compared to those treated with external fixation alone, highlighting the need for careful patient selection and optimization of treatment protocols. Further prospective studies are warranted to clarify the optimal indications and techniques for the combined approach in this specific patient population and to develop standardized guidelines to improve treatment outcomes and reduce complications.

Given the challenges associated with the combined treatment strategies in managing pelvic fractures with abdominal organ injury, future research should aim to refine protocols and improve coordination of care (81, 82). Prospective multicentric studies comparing organ-specific outcomes (e.g., hepatic vs. splenic injury management) are crucial in establishing best practices. For example, Zheng et al. (72) proposed a risk stratification model integrating injury severity scores (ISS) and organ-specific parameters (e.g., AAST grading for splenic injuries) to guide embolization timing. Additionally, exploring innovative imaging technologies, such as dual-phase CT angiography, may improve early detection of abdominal vascular injuries and reduce diagnostic delays (42). Concurrently, further investigation into tailored approaches for different age groups and comorbid conditions may promote a personalized strategy that optimally balances intervention risks with benefits. The integration of artificial intelligence (AI) for real-time decision support in trauma resuscitation, as proposed by Ahmed et al. (76), could enhance interdisciplinary coordination and procedural timing.

## 8 Conclusion

Addressing pelvic fractures with associated abdominal organ injuries requires a collaborative approach among trauma surgeons, interventional radiologists, and other healthcare professionals, as evidenced by varied outcomes across studies. The integration of innovative techniques, such as biodegradable materials and advanced imaging, holds promise for enhancing treatment efficacy. Future research should focus on standardizing protocols and refining training for trauma teams to optimize patient care, ultimately improving recovery rates and reducing complications in this complex field of medicine.
